# Plasma selenium levels and nonalcoholic fatty liver disease in Chinese adults: a cross-sectional analysis

**DOI:** 10.1038/srep37288

**Published:** 2016-11-17

**Authors:** Zhen Yang, Chonghuai Yan, Gang Liu, Yixin Niu, Weiwei Zhang, Shuai Lu, Xiaoyong Li, Hongmei Zhang, Guang Ning, Jiangao Fan, Li Qin, Qing Su

**Affiliations:** 1 Department of Endocrinology, Xinhua Hospital, Shanghai Jiao Tong University School of Medicine, Shanghai, China; 2 Ministry of Education-Shanghai Key Laboratory of Children’s Environmental Health, Xinhua Hospital, Shanghai Jiao Tong University School of Medicine, Shanghai, China; 3 Key Laboratory of Nutrition and Metabolism, Institute for Nutritional Sciences, Shanghai Institutes for Biological Sciences, Chinese Academy of Sciences, University of Chinese Academy of Sciences, Shanghai, China; 4 Department of Endocrinology, Xinhua Hospital Chongming Branch, Shanghai Jiao Tong University School of Medicine, Shanghai, China; 5 Department of Endocrinology and Metabolism, Key Laboratory for Endocrine and Metabolic Diseases of Ministry of Health, RuiJin Hospital, Shanghai Jiao Tong University School of Medicine, E-Institute of Shanghai Universities, Shanghai, China; 6 Department of Gastroenterology, Shanghai Key Laboratory of Children’s Digestion and Nutrition, Xinhua Hospital, Shanghai Jiaotong University School of Medicine, Shanghai, China

## Abstract

Selenium exposure can induce liver insulin resistance and increased liver triglyceride concentrations in animals, which may link to an increased risk of nonalcoholic fatty liver disease (NAFLD). However, epidemiological studies investigating the association between elevated plasma selenium levels and NAFLD were not available. We aimed to investigate the association of selenium levels with the prevalence of NAFLD in Chinese adults. This was a cross-sectional study of 8550 Chinese adults aged 40 yr or older in Shanghai, China. A questionnaire, anthropometric measurements, and laboratory tests were conducted. NAFLD was diagnosed by hepatic ultrasound after the exclusion of alcohol abuse and other liver diseases. Plasma selenium concentration was assessed by inductively coupled plasma mass spectroscopy. The median concentration of plasma selenium was 213.0 μg/L. Elevated plasma selenium levels were associated with higher triglycerides, LDL-cholesterol, fasting plasma glucose, post-loading plasma glucose, A1c, HOMA-IR, as well as ALT, AST and γ-GT (all P < 0.05). The odds ratios were substantially higher for NAFLD (OR = 1.54, 95% CI 1.13–2.18) in the highest selenium quartile compared with those in the lowest quartile, after adjustment for potential cofounder. The results of this study provided epidemiological evidence that increased plasma selenium level is associated with elevated prevalence of NAFLD.

Nonalcoholic fatty liver disease (NAFLD) is characterized by excessive hepatic fat accumulation of patients who have no history of alcohol abuse^1^. NAFLD is strongly linked to insulin resistance, type 2 diabetes and obesity, being prevalent in up to 95% of obese patients and up to 70% of people with type 2 diabetes^2^. It is reported that NAFLD affects about 30% of the general population in Western countries^3^. The prevalence of NAFLD is increasing in China because of the westernization of the lifestyle, such as a high-fat and high-calorie diet and less physical activity. A recent epidemiological study revealed that in a Chinese population, the prevalence of NAFLD is 23.3%^4^, which indicates that not only in the Western population, but also in the relatively leaner Chinese population, NAFLD is highly epidemic. Although excess energy intake and sedentary lifestyle are well-recognized risk factors for NAFLD, growing evidence has suggested that environmental exposures may contribute to the pathogenesis of NAFLD^5^,^6^,^7^,^8^,^9^.

As an essential trace element in human nutrition, selenium is widely distributed in nature in most rocks, soils and sediment. Most processed selenium is used in the electronics industry, glass industry and as a component of pigments in plastics, paints, enamels, inks, and rubber^10^,^11^,^12^. Moreover, selenium can be released into ambient air and soil when burning coal, fuel oil and waste or discharging sewage^13^,^14^,^15^,^16^,^17^. Therefore, the general public are exposed to selenium from air, foods and drinking water^18^,^19^,^20^,^21^. Other sources of selenium exposure may come from use of selenium nutritional supplementation and antidandruff shampoos^22^,^23^,^24^. Most of the selenium that enters the body quickly leaves the body, usually within 24 hours^25^. Beyond what the body needs, selenium leaves mainly in the urine, but also in feces and breath. Selenium can build up in the human body, however, if exposure levels are very high or if exposure occurs over a long time. The amount that builds up in the body depends on the chemical form of the selenium. It builds up mostly in the liver and kidneys but also in the blood, lungs, heart, and testes^26^.

The trace mineral selenium is essential for human health. Previous studies have demonstrated selenium play a pivotal role in redox homeostasis, thyroid hormone metabolism, and protection from oxidative stress and inflammation^27^. Some canonical medical guidance are suggesting people to use the selenium as a dietary supplement daily for preventing cell-damage from the free radicals^27^. However, more recently, findings from observational epidemiological studies and randomized clinical trials have raised concern that high selenium exposure may lead to metabolism abnormalities, including dyslipidemia, type 2 diabetes or insulin resistance^28^.

To date, it remains largely unclear whether elevated circulating level is associated with NAFLD risk in humans. Several animal studies have indicated that selenium exposure can induce increased serum liver enzyme levels, activation of Kupffer cells, higher liver insulin resistance and higher liver triglyceride concentrations than controls^29^,^30^,^31^. Therefore, evidence from animals suggest that selenium exposure may be associated with the developing of NAFLD. However, evidence from human studies is scarce regarding whether selenium exposure is associated with NAFLD. In this study, we investigated the levels of plasma selenium in a Chinese population and analyzed its association with NAFLD.

## Results

The median concentration of plasma selenium was 213 μg/L (interquartile range: 181.6–247.4 μg/L) in this study. As shown in Table 1, participants with higher plasma selenium concentrations were more likely to be current smokers (*P* < 0.01) and higher waist circumference (*P* < 0.01), systolic blood pressure (*P* < 0.01). Furthermore, participants with increased plasma selenium concentration tended to have elevated levels of triglycerides, low-density lipoprotein-cholesterol, fasting plasma glucose, post-loading plasma glucose, HbA1c, and HOMA-IR, as well as ALT, AST and γ-GT (all *P* for trend < 0.05) (Table 1).

**Figure 1:**
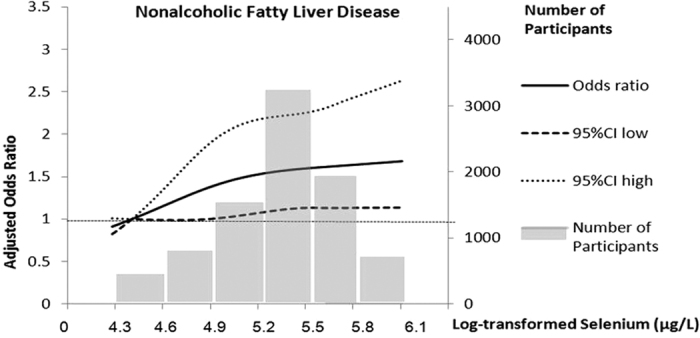
Odds ratio of nonalcoholic fatty liver diseases by log-transformed plasma selenium concentrations. Lines represent odds ratios (95% CI) based on restricted cubic splines for log-transformed plasma selenium concentrations with knots at the 5th, 50th and 95th percentiles. Odds ratios were estimated using a logistic regression model after adjustment for age, gender, BMI, current smoking status, physical activity, waist circumference, systolic blood pressure, diastolic blood pressure, fasting plasma glucose, post-loading plasma glucose, HOMA-IR, lipid profiles, estimated glomerular filtration rate and C-reactive protein; P for linear <0.01. Bars represent the numbers of participants, 6 equally sized bins were selected from the 1st to the 99th percentiles of log-transformed selenium distribution.

Compared with those without NAFLD, participants with NAFLD had elevated plasma selenium concentration (median: 270.2 μg/L in NAFLD vs 192.5 μg/L in non-NAFLD subjects, P < 0.01) (Table 2). The ORs (95% CIs) for NAFLD from the lowest to the highest plasma selenium quartiles were 1.29 (0.99–1.77), 1.79 (1.26–2.37) and 1.60 (1.17–2.18), respectively (referencing to 1.00) (P for trend < 0.001) (Table 3), after adjusting for age, gender (model 1). The selenium-NAFLD association was not materially changed (P for trend < 0.001) by further controlling for lifestyle covariates (model 2), as well as additionally adjusting for waist circumference, systolic blood pressure, diastolic blood pressure, fasting plasma glucose, post-loading plasma glucose, HOMA-IR, lipid profiles and estimated glomerular filtration rate (log-transformed) (model 3) and liver enzyme profiles and CRP (model 4) (all P for trends <0.001). A positive log-linear dose–response relationship was evident in the cubic spline regression model (Fig. 1, *P* < 0.01 for linearity).

**Figure 2:**
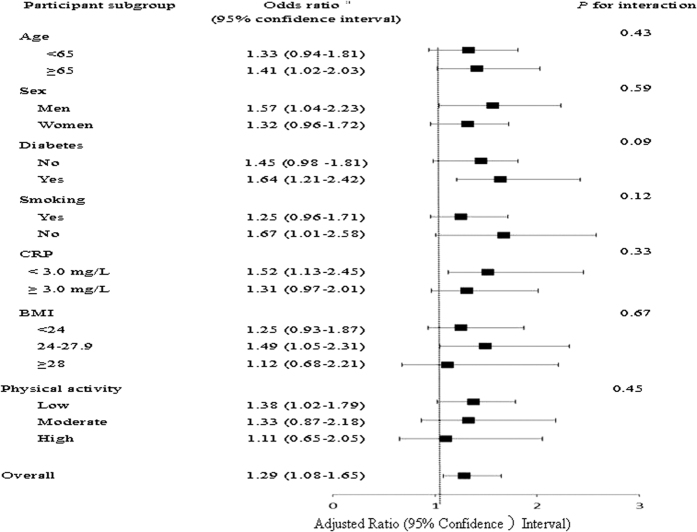
Stratified analyses of the associations [odds ratio (95% confidence interval)] between plasma selenium concentrations and nonalcoholic fatty liver diseases. ^a^Adjusted for age, gender, BMI, current smoking status, physical activity, waist circumference, systolic blood pressure, diastolic blood pressure, fasting plasma glucose, post-loading plasma glucose, HOMA-IR, lipid profiles, estimated glomerular filtration rate and C-reactive protein, stratifying factors excepted.

When plasma selenium concentration was considered as a continuous variable, the overall OR (95% CI) of having NAFLD was 1.29 (1.08–1.65) per unit increment of log-transformed selenium concentration. In the stratified analyses, the selenium-NAFLD association was slightly stronger in non-smokers and individuals with lower physical activity levels as compared with their counterparts (Fig. 2). However, no interaction was detected with any of the variables (all *P* for interaction > 0.10).

## Discussion

To our knowledge, this is the first population-based study showing that elevated plasma selenium concentrations were associated with an increased risk of having NAFLD. The association was independent of traditional NAFLD risk factors including lifestyle, BMI, lipid profiles and inflammatory biomarkers.

China has areas of both selenium deficiency and excess^27^. The geochemistry and human health impacts of trace elements selenium have been intensively studied in China, in terms of geochemical sources, distribution, and health impact^32^. The diseases (including Keshan disease and Kashin-Beck disease) were related to the deficiency of selenium in the low-selenium geological belt with selenium contents in soil stretching from northeast to southwest of China, while southeast of China in the rich-selenium geological belt^32^. The sample collection area of this study located in southeast of China. The median concentration of plasma selenium in our population was 213 μg/L. In most previously published studies, plasma selenium values, however, varied from 41 to 210 μg/L among people who living in New Zealand, Canada, Finland, Italy, South Africa and USA^33^,^34^,^35^,^36^,^37^,^38^. Currently, there is no internationally acceptable value or range for plasma selenium concentration in the general population. Thus, it remains to be elucidated whether or to what extent the discrepancies regarding plasma selenium concentrations could be explained by its exposure levels, effects of genetic predisposition and other predisposing factors on its metabolism^39^, between-laboratory differences in methods (ICP-MS vselectrothermal atomic absorption spectrometry) and measurement errors, or variations in population characteristics among studies.

For the general population, the primary exposure pathways, in order of decreasing relative proportions, are food, water, and air^39^. Some studies found high amounts of selenium in foods like nuts, seafood, meats and wheat^40^. However, we did not observe correlations between plasma selenium concentrations and consumption of rice, wheat or seafood (data not shown). In addition, tobacco smoking is another small but important determinant of selenium status^40^. Previous studies have demonstrated smokers had lower tissue selenium concentrations than did nonsmokers^41^,^42^. In line with this idea, we found that both the median concentration of plasma selenium and the OR for having NAFLD were lower in smokers than in their non-smoking counterparts (Fig. 2). Certainly, more studies are needed to clarify the major sources of selenium exposure and its health outcomes in different populations.

In our study, elevated levels of plasma selenium were associated with not only elevated prevalence of NAFLD, but also increased levels of fasting plasma glucose, post-loading plasma glucose, HbA1C, HOMA-IR, triglycerides, ALT, AST and γ-GT. Limited data suggest that hepatotoxicity can occur in humans following acute oral exposure to sodium selenate, but no definitive studies were located regarding hepatic effects in humans after intermediate or chronic oral exposure to selenium compounds. Tests following an acute poisoning of a 15-year-old girl with sodium selenate revealed abnormally elevated serum bilirubin and alkaline phosphatase^43^. However, hepatic effects, such as changes in serum liver enzymes or liver morphology (identified by ultrasonography), have not been observed in humans at chronic dietary intakes of selenium^44^,^45^. Therefore, more large-scale population-based studies are needed to clarify the role of selenium exposure in the pathogenesis of NAFLD in the future. Evidence from studies in rodent models demonstrated that selenium exposure was more potent in inducing liver damage by activating inflammation and the liver with infiltration by inflammatory cells, increasing hepatic enzymes and accumulation of glycogen and lipid^29^,^30^,^31^.

The underlying mechanism of selenium exposure in the pathogenesis of NAFLD is not yet fully elucidated. SeP (in humans encoded by the SEPP1 gene), a secretory protein primarily produced by the liver^46^, contains 10 selenocysteine residues and functions as a selenium transporter^47^. Recently, Misu *et al.*^48^ found a positive correlation between hepatic SEPP1 mRNA levels and insulin resistance in humans. Administration of purified SeP impaired insulin signaling and dysregulated glucose metabolism both *in vitro* and *in vivo*. In contrast, genetic deletion and RNA interference-mediated knockdown of SeP improved systemic insulin sensitivity and glucose tolerance in mice. Moreover, Mueller *et al.* found that selenium could also raise liver PTP1b activity in rats, which might lead to further deterioration of insulin sensitivity in liver^31^. In line with views, we observed that HOMA-IR increased with plasma selenium quartiles in this study (*P* < 0.001), which indicate that high circulating selenium levels is correlated with impaired insulin signaling and could potentially modulate liver insulin resistance. It is well-known that insulin resistance plays a pivotal role in the development of hepatic lipid accumulation. Furthermore, some studies have also observed plasma triglyceride levels in the animals model were increased by the high dietary selenium intake^31^,^49^, which indicate supranutritional selenium could induce alterations in molecular targets related to energy metabolism in skeletal muscle and visceral adipose tissue^50^. Taken together, these important and intriguing results suggest that high selenium may play a causal role in the pathogenesis of liver fat accumulation. However, whether these findings in animal models can explain the association of selenium exposure with NAFLD in humans needs thorough investigations in the future.

To our knowledge, this is the first relatively large-scale population study that has revealed the association of elevated plasma selenium levels with NAFLD. In addition, we have measured a wide spectrum of lifestyle and biochemical risk factors, which allow a careful control for the potential confounding effects in the analyses.

There are several limitations to be considered. First, due to the cross-sectional nature, a causal relationship between selenium and NAFLD cannot be established. There is a possibility that in the condition of some pathological metabolism, such as NAFLD, the selenium could be under retention. Thus, it is critical to carry out prospective studies in the future. Second, ultrasonographic examination was used to determine the presence of NAFLD. However, the sensitivity of liver ultrasonography may vary depending on the hepatic fat content^51^, although as discussed above, liver ultrasonography offers several strengths including the non-invasive nature of the test. In addition, although the sensitivity of liver ultrasonography may vary with the hepatic fat content, when performed properly, ultrasonography has been reported to detect as little as ≥5% hepatic fat content^51^. Furthermore, several advantages of ultrasound imaging, including portability, low cost, and simplicity of use, make it further applicable and acceptable for investigating the incidence, prevalence, and risk factors of NAFLD in large-scale populations, particularly in developing countries. Third, given the diagnosis of NAFLD in the present study was based on ultrasound imaging, which means that NAFLD patients in our study were in at least moderate stage of the disease. Therefore, we failed to assess the association between plasma selenium and mild-stage NAFLD in the present study. Finally, it is noteworthy that other environmental confounding factors may affect our conclusions and such factors, if discovered, need to be taken into account in future analyses.

In summary, our study showed for the first time that elevated plasma selenium concentrations were associated with increased prevalence of NAFLD in a Chinese population. From the perspective of public health, it is interesting and important to confirm whether there is a causal role of selenium exposure during the pathogenesis of NAFLD in humans. Therefore, more studies in the general population, particularly with prospective designs, are warranted. Studies are also needed to elucidate the potential mechanisms underlying the relation between elevated plasma selenium levels and NAFLD in humans.

## Methods

### Study participants and design

In 2011 China a national survey of Risk Evaluation of cAncers in Chinese diabeTic Individuals: a lONgitudinal (REACTION) study, which was conducted among 259,657 adults, aged 40 years and older in 25 communities across mainland China, from 2011 to 2012^52^. The data presented in this article are based on the baseline survey of subsamples from Shanghai in eastern China. All studied individuals came from the Chongming District in Shanghai, China. There were 9930 participants who had complete information about age; sex; smoking and alcohol consumption habits; and a medical history including the use of medications, BMI, and a hepatic ultrasonic examination. Participants meeting the following criteria were excluded: 1) those with a history of known liver diseases such as hepatitis, cirrhosis, or malignancy; 2) those with more than 3 times the normal serum alanine aminotransferase (ALT), aspartate aminotransferase (AST) or γ-glutamyltransferase (GGT) levels of the study population; and 3) those with alcohol consumption greater than 140 g/wk for men and 70 g/wk for women. Thus, a total of 8550 participants (2739 men and 5811 women) were eventually included in this analysis (Table 2). The study protocol was approved by the Ethics Committee of Xinhua Hospital Affiliated to Shanghai Jiaotong University School of Medicine, and all studies were carried out in accordance with the approved guidelines. Written informed consent was obtained from all the participants.

### Data collection

Subjects were admitted after an overnight fast of 10 h and underwent a 75-g OGTT. The fasting and OGTT 2-h venous blood samples were collected into a routine tube, respectively, and were immediately processed by centrifugation at 4 °C for 10 min at 3000 relative centrifugal force. Fasting plasma glucose, post-loading plasma glucose, fasting and post-loading serum insulin concentrations, lipids profile including triglycerides, high-density lipoprotein cholesterol, low-density lipoprotein cholesterol, alanine aminotransferase, aspartate aminotransferase, and γ-glutamyltranspeptidase were detected within 1 h of collection. Another anticoagulated venous blood (heparin) was collected for measurement of hemoglobin A1c within 4 h of collection. The smoking was defined as never, current (smoking regularly in the past 6 months), or ever (cessation of smoking for more than 6 months). Physical activity was estimated using the short form of the International Physical Activity Questionnaire by adding questions on frequency and duration of moderate and vigorous activities and walking (Guidelines for data processing and analysis of the International Physical Activity Questionnaire (IPAQ). Available at: http://www.ipaq.ki.se/ (2006)).

Venous plasma glucose level was measured by glucose oxidase method (ADVIA-1650 Chemistry System, Bayer, Leverkusen, Germany), hemoglobin A1c was measured by high-performance liquid chromatography (BIO-RAD, D10, CA). Fasting insulin was determined by RIA (Linco Research, St. Charles, MO). Serum C-reactive protein was determined by ELISA with Duoset kit (R&D Systems, Minneapolis, MN). Serum creatinine, triglycerides, total cholesterol, high-density lipoprotein cholesterol, low-density lipoprotein cholesterol, alanine aminotransferase, aspartate aminotransferase, and γ-glutamyltranspeptidase were measured with an autoanalyzer (Hitachi 7080; Tokyo, Japan). The homeostasis model assessment of insulin resistance (HOMA-IR) was calculated according to the equation described by Matthews *et al.*^53^. The abbreviated Modification of Diet in Renal Disease formula recalibrated for Chinese was used to estimate glomerular filtration rate expressed in milliliters per minute per 1.73 m^2^: estimated glomerular filtration rate (eGFR) = 186 × [serum creatinine × 0.011]^−1.154^ × [age]^−0.203^ × [0.742 if female] × 1.233, where serum creatinine is expressed as micromoles per liter and 1.233 is the adjusting coefficient for Chinese^54^.

### Measurement of plasma selenium concentration

Fasting peripheral venous blood samples were collected by EDTA-contained tubes and centrifuged to separate plasmathen stored at −80 °C until analysis. Each 200 μl of the plasma samples was mixed with 2 ml 2% HNO3. The mixtures were injected into an Agilent 7500ce inductively coupled plasma mass spectroscopy system (ICP-MS, Agilent Technologies, Tokyo, Japan). All the containers or tubes were pre-cleaned by overnight soaking in ultrapure grade 2% HNO3 solution. Quality control was performed (1 out of 20 samples), and the inter- and intra-assay coefficients of variation were <10% and <8%, respectively. All participants had plasma selenium levels above the detection limit (0.2526 μg/L).

### Definition of NAFLD

Hepatic ultrasonic examination was performed on all participants by two trained ultrasonographists who were blinded to the clinical and laboratory data, using a high-resolution B-mode tomographic ultrasound system (EsaoteBiomedicaSpA, Italy) with a 3.5-MHz probe. Diagnosis of fatty liver by ultrasonography was defined by the presence of at least two of three abnormal findings: diffusely increased echogenicity of the liver relative to the kidney, ultrasound beam attenuation, and poor visualization of intrahepatic structures^1^. NAFLD was diagnosed by hepatic ultrasound after the exclusion of alcohol abuse and other liver diseases^55^,^56^.

### Statistical analysis

Analysis of covariance for continuous variables and multivariate logistic regression analysis for categorical variables were applied for the comparison across plasma selenium quartiles. Whenever appropriate, log10 transformations of skewed variables were used in analyses. A logistic regression model was used to test odds ratios (ORs) and confidence intervals (CIs) of NAFLD for each plasma selenium quartile compared with the lowest quartile, with adjustment for age (continuous), gender, current smoking status (yes, no), physical activity (low, moderate, high), BMI (continuous), waist circumference, systolic blood pressure, diastolic blood pressure, fasting plasma glucose, post-loading plasma glucose, HOMA-IR, lipid profiles, and estimated glomerular filtration rate (log-transformed continuous variable).

Tests of linear trend across increasing selenium quartiles were conducted by assigning the median value to each quartile and treating it as a continuous variable. Plasma CRP was further adjusted to test influence of inflammatory status on the association. The log-linear dose-response relationship was estimated by applying a restricted cubic spline regression model with 3 knots at the 5th (140 μg/L), 50th (213 μg/L) and 95th (300 μg/L) percentiles. Stratified analyses were performed according to age (<65, ≥65), sex, current smoking status, diabetes, CRP levels, BMI category (normal weight, overweight and obesity) and physical activity. Likelihood ratio tests were conducted to examine interactions. All statistical analysis were performed with SAS (version 9.3; SAS Institute Inc., Cary, NC). P values < 0.05 were considered statistically significant.

## Additional Information

**How to cite this article**: Yang, Z. *et al.* Plasma selenium levels and nonalcoholic fatty liver disease in Chinese adults: a cross-sectional analysis. *Sci. Rep.* **6**, 37288; doi: 10.1038/srep37288 (2016).

**Publisher’s note:** Springer Nature remains neutral with regard to jurisdictional claims in published maps and institutional affiliations.

## Figures and Tables

**Table 1:** Characteristics of participants according to plasma selenium quartiles.

	Q1: <181.6 μg/L	Q2: 181.6–213.0 μg/L	Q3: 213.0–247.4 μg/L	Q4: >247.4 μg/L	P for trend
Selenium (mg/l)	0.16 (0.15–0.17)	0.20 (0.19–0.21)	0.23 (0.22–0.24)	0.27 (0.26–0.30)	<0.001
Age (years)	56.1 ± 8.0	55.8 ± 8.0	56.1 ± 7.8	56.3 ± 7.9	0.26
Female	(62.8)	(69.5)	(66.2)	(64.2)	0.35
Current smokers	(15.6)	(12.8)	(14.1)	(19.9)	<0.001
Physical activity					0.97
Low	(72.5)	(71.4)	(70.2)	(73.1)	
Moderate	(21.4)	(19.8)	(20.3)	(19.4)	
High	(6.1)	(8.8)	(9.5)	(7.5)	
Body mass index (kg/m^2^)	24.7 ± 3.5	24.5 ± 3.4	24.3 ± 3.2	24.8 ± 3.8	0.27
Waist circumference (cm)	83.2 ± 9.5	83.3 ± 9.5	83.7 ± 9.6	84.9 ± 9.8	0.005
Systolic blood pressure (mmHg)	129.5 ± 20.3	131.8 ± 17.8	130.2 ± 17.9	134.5 ± 21.2	0.001
Diastolic blood pressure (mmHg)	81.8 ± 10.8	81.0 ± 10.6	80.1 ± 10.8	81.2 ± 11.1	0.28
Triglycerides (mmol/l)	1.32 (0.91–1.84)	1.34 (1.00–2.10)	1.47 (1.01–2.22)	1.57 (1.07–2.39)	<0.001
Total cholesterol (mmol/l)	4.89 ± 0.92	4.72 ± 0.99	4.88 ± 0.98	4.94 ± 0.97	0.01
High-density lipoprotein cholesterol (mmol/l)	1.29 ± 0.32	1.27 ± 0.32	1.30 ± 0.31	1.28 ± 0.31	0.76
Low-density lipoprotein cholesterol (mmol/l)	2.63 ± 0.75	2.69 ± 0.78	2.73 ± 0.76	2.73 ± 0.76	0.034
Fasting plasma glucose (mmol/l)	6.23 ± 1.35	6.28 ± 1.95	6.41 ± 1.90	6.71 ± 2.25	<0.001
Post-loading plasma glucose (mmol/l)	8.52 ± 3.59	8.72 ± 3.67	8.93 ± 3.96	9.57 ± 4.32	<0.001
Hemoglobin A1c (%)	5.6 (5.3–6.0)	5.9 (5.5–6.4)	6.1 (5.7–6.5)	6.2 (5.8–6.7)	<0.001
HOMA-IR	1.66 (1.19–2.50)	1.68 (1.21–2.58)	1.74 (1.27–2.65)	1.82 (1.29–2.90)	<0.001
C-reactive protein (mg/l)	1.37 (0.55–3.41)	1.49 (0.62–3.67)	1.55 (0.64–3.65)	1.43 (0.53–3.39)	0.57
eGFR (ml/min per 1.73 m^2^)	121.1 ± 25.0	122.2 ± 24.2	120.6 ± 20.5	121.8 ± 22.9	0.34
ALT (U/L)	14 (10–20)	15 (11–20)	16 (12–21)	17 (12–23)	<0.001
AST (U/L)	20 (16–24)	21 (17–25)	21 (18–26)	22 (19–27)	<0.001
γ-GT (U/L)	19 (13–31)	20 (14–32)	21 (14–34)	22 (15–40)	<0.001

Abbreviations: Q, quartile; HOMA-IR, homeostasis model assessment of insulin resistance; eGFR, estimated glomerular filtration rate; ALT, alanine aminotransferase; AST, aspartate aminotransferase; γ-GT, γ-glutamyltranspeptidase.

Data are means ± s.d. or medians (interquartile ranges) or numbers (proportions).

**Table 2:** Clinical and laboratory characteristics of NAFLD and control subjects.

	Without NAFLD group (n = 5118)	NAFLD group (n = 3732)	P value
Age (years)	55.4 ± 7.9	57.0 ± 7.3	<0.001
Sex (male/female)	1749/3069	990/2742	<0.001
Systolic blood pressure (mmHg)	128.4 ± 19.3	133.6 ± 18.5	<0.001
Diastolic blood pressure (mmHg)	79.1 ± 10.3	82.0 ± 10.0	<0.001
Waist circumference (cm)	81.2 ± 10.3	89.2 ± 8.6	<0.001
Body mass index (kg/m^2^)	23.3 ± 3.0	26.4 ± 6.2	<0.001
Triglycerides (mmol/l)	1.1 (0.9–1.6)	1.8 (1.4–2.6)	<0.001
Total cholesterol (mmol/l)	4.6 ± 0.9	4.8 ± 1.1	<0.001
High-density lipoprotein cholesterol (mmol/l)	1.29 ± 0.33	1.14 ± 0.27	<0.001
Low-density lipoprotein cholesterol (mmol/l)	2.54 ± 0.76	2.69 ± 0.80	<0.001
Fasting plasma glucose (mmol/l)	6.0 ± 1.5	6.6 ± 2.0	<0.001
Post-loading plasma glucose (mmol/l)	7.8 ± 3.3	9.8 ± 4.3	<0.001
HbA1c (%)	5.8 ± 0.8	6.2 ± 1.0	<0.001
HOMA-IR	1.5 (1.1–1.9)	2.5 (1.8–3.4)	<0.001
C-reactive protein (mg/l)	1.45 (0.56–3.47)	1.51 (0.63–3.79)	<0.001
eGFR (ml/min per 1.73 m^2^)	124.9 (110.6–140.3)	121.3 (106.9–135.8)	<0.001
ALT (U/l)	14.3 ± 10.1	21.4 ± 15.6	<0.001
AST (U/l)	19.3 ± 8.8	22.5 ± 12.3	<0.001
γ-GT (U/l)	24.0 ± 28.1	37.2 ± 48.3	<0.001
Selenium (μg/L)	192.5 (182.3–203.9)	270.2 (256.4–288.5)	<0.001

Abbreviations: HOMA-IR, homeostasis model assessment of insulin resistance; eGFR, estimated glomerular filtration rate; ALT, alanine aminotransferase; AST, aspartate aminotransferase; γ-GT, γ-glutamyltranspeptidase.

Data are means ± s.d. or medians (interquartile ranges) or numbers (proportions).

**Table 3:** Odds ratio (95% confidence interval) of nonalcoholic fatty liver disease according to quartiles of plasma selenium concentrations.

	Quartile 1	Quartile 2	Quartile 3	Quartile 4	P for trend
	<181.6 μg/L	181.6–213 μg/L	213.1–247.4 μg/L	>247.4 μg/L	
No. cases/participants	613/2212	809/2213	1145/2212	1165/2213	
Model 1^a^	1	1.29 (0.99–1.77)	1.79 (1.26–2.37)	1.60 (1.17–2.18)	<0.001
Model 2^b^	1	1.29 (0.98–1.78)	1.75 (1.22–2.34)	1.58 (1.15–2.18)	<0.001
Model 3^c^	1	1.24 (0.96–1.77)	1.70 (1.16–2.32)	1.52 (1.11–2.14)	<0.001
Model 4^d^	1	1.27 (0.95–1.77)	1.72 (1.19–2.33)	1.54 (1.13–2.18)	<0.001

^a^Model 1: adjusted for age, gender.

^b^Model 2: additionally adjusted for BMI, current smoking status, drinking status, physical activity.

^c^Model 3: additionally adjusted for waist circumference, systolic blood pressure, diastolic blood pressure, fasting plasma glucose, post-loading plasma glucose, HOMA-IR, lipid profiles and estimated glomerular filtration rate.

^d^Model 4: additionally adjusted for liver enzyme profiles and C-reactive protein.
